# Molecular Mechanisms Responsible for the Rescue Effects of Pamidronate on Muscle Atrophy in Pediatric Burn Patients

**DOI:** 10.3389/fendo.2019.00543

**Published:** 2019-08-07

**Authors:** Fabrizio Pin, Andrea Bonetto, Lynda F. Bonewald, Gordon L. Klein

**Affiliations:** ^1^Department of Anatomy and Cell Biology, Indiana University School of Medicine, Indianapolis, IN, United States; ^2^Indiana Center for Musculoskeletal Health, Indiana University School of Medicine, Indianapolis, IN, United States; ^3^Department of Surgery, Indiana University School of Medicine, Indianapolis, IN, United States; ^4^Simon Cancer Center, Indiana University School of Medicine, Indianapolis, IN, United States; ^5^Department of Otolaryngology—Head and Neck Surgery, Indiana University School of Medicine, Indianapolis, IN, United States; ^6^Department of Orthopaedic Surgery, Indiana University School of Medicine, Indianapolis, IN, United States; ^7^Department of Orthopaedic Surgery, University of Texas Medical Branch, Galveston, TX, United States

**Keywords:** burn, muscle wasting, pamidronate, TGFβ, muscle catabolic factors

## Abstract

Not only has pamidronate been shown to prevent inflammation associated bone resorption following burn injury, it also reduces protein breakdown in muscle. The aim of this study was to identify the molecular mechanisms responsible for muscle mass rescue in pamidronate treated compared to placebo/standard of care-treated burn patients. Mature myotubes, generated by differentiating murine C2C12 myoblasts, were exposed for 48 h to 1 or 5% serum obtained from 3 groups of children: normal unburned, burned receiving standard of care, and burned receiving standard of care with pamidronate. Exposure to serum from burned patients caused dose-dependent myotube atrophy compared to normal serum as expected based on previous observations of muscle atrophy induced by burn injury in humans and animals. The size of C2C12 myotubes was partially protected upon exposure to the serum from patients treated with pamidronate correlating with the rescue of muscle size previously observed in these patients. At the molecular signaling level, serum from both pamidronate and non-pamidronate-treated burn patients increased pSTAT3/STAT3 and pERK1/2/ERK1/2 compared to normal serum with no significant differences between the two groups of burn patients indicating elevated production of inflammatory cytokines. However, serum from pamidronate-treated patients restored the phosphorylation of AKT and mTOR and reduced protein ubiquitination when compared to burn serum alone, suggesting a prevention of muscle catabolism and a restoration of muscle anabolism. Myotube atrophy induced by burn serum was partially rescued after exposure to a pan anti-TGFβ-1/2/3 antibody, suggesting that this signaling pathway is partially responsible for the atrophy and that bisphosphonate protection of bones from resorption during burn injury prevents the release of muscle pro-catabolic factors such as TGFβ into the circulation.

## Introduction

Unintentional burn injury in the pediatric population is one of the common causes of mortality and morbidity, representing the fourth cause of death in the United States according to the World Health Organization (1). The total cost of unintentional pediatric burn injuries was estimated at 2.1 billion dollars (2).

Burn injury, in addition to damaging the site of burn, can produce a systemic response, especially when the injured skin surface covers >20% of the total body surface area (3). The extensive thermal injury is accompanied by acute catabolism resulting in lean mass and muscle wasting, negative nitrogen balance, and bone resorption. Other important long-term risks can be growth delay and increased risk of fracture (4, 5).

Among the underlying adaptive responses to burn injury that contribute to this catabolic response are the systemic inflammatory response resulting in increased inflammatory cytokine production, such as interleukin (IL)-1 and IL-6 and the acute stress response, resulting in the increased production of endogenous glucocorticoids (4, 6). Muscle wasting results in a negative muscle protein balance, reduction of muscle mass followed by a functional deficit. This event begins acutely but is sustained over the first year post-burn and can impair rehabilitation compromising the recovery (7). During the acute phase post-burn, the balance between protein synthesis and degradation is impaired, leading to skeletal muscle atrophy (8). This alteration seems to be predominantly mediated by the hyperactivation of the ATP-ubiquitin-proteasome system through the inflammatory response (9). Bone resorption begins on the first day post-burn (10) and continues over the first 2 weeks resulting in a loss of up to 7% lumbar spine bone mineral content and density by 3 weeks post-burn (11). Up to 3% of total body bone mineral content is lost by 6 months post-burn (11). The incidence of post-burn fractures is elevated in children and estimated at 15% (12).

In a previous publication, Klein et al. (11) found that a single administration of the nitrogen-containing bisphosphonate pamidronate within 10 days of the burn injury eliminated resorptive bone loss and preserved bone density in a randomized, double- blinded, placebo-controlled study of severely burned children. Bisphosphonates are anti-resorptive agents which accumulate in bone matrix, and on resorption, are taken up directly by osteoclasts. They inhibit the enzyme farnesyl pyrophosphate synthase (FPS), directly interfering in cholesterol biosynthesis and impairing cell membrane integrity and signal transduction. The result is osteoclast apoptosis and inhibition of bone resorption (13). Przkora et al. (14) found that this rescue effect lasted at least 2 years. In addition, Borsheim et al. (15) described a decrease in muscle protein breakdown, an increase in lower extremity muscle fiber diameter and muscle strength, and a net positive muscle protein balance in burned children who received the single dose of pamidronate compared to a placebo. While the preservation of bone mass was expected due to the affinity of bisphosphonate for bone, the effect of bisphosphonate on muscle protein balance was unanticipated. Therefore, we undertook a study to investigate possible mechanisms by which bisphosphonates may have effected these changes in muscle. A candidate mechanism for investigation is the transforming growth factor (TGF)β production, which has been shown to be elevated in the serum of burned patients (16) and burned animals (17, 18), associated with both hypertrophic scarring (16) and immunosuppression (17, 18). Furthermore, Waning et al. (19) have demonstrated that TGFβ released following bony metastases in breast cancer affects the ryanodine receptor in muscle, causing a calcium leak and cachexia. This effect was inhibited by treatment with bisphosphonates.

## Materials and Methods

### Cell Culture

Murine C2C12 skeletal myoblasts (ATCC, Manassas VA) were grown as described previously (15) in high-glucose DMEM supplemented by 10% FBS, 100 U/ml penicillin, 100 μg/ml streptomycin, 100 μg/ml sodium pyruvate, 2 mmol L-glutamine, and maintained at 37°C in 5% CO_2_ in air. Differentiation of myotubes was induced by switching subsonfluent myoblasts to differentiation medium DMEM supplemented by 2% horse serum. The differentiation medium was replaced every other day for up to 5 days. Fully differentiated C2C12 myotubes were exposed for 48 h to 1 or 5% serum from patients enrolled in a randomized controlled trial of pamidronate. In order to determine the effect of transforming growth factor (TGF)-β pan neutralizing antibody on fiber size myotubes were exposed for 48 h to 10 μg/ml of anti-TGFβ-1/2/3, clone 1D11.16.8 (BioXcell West, Lebanon NH) in the presence or absence of burned patients' serum.

### Patients

Three different types of serum were used in the study: that from normal, unburned children (N) (*n* = 5), severely burned children given placebo in the randomized trial (B), *n* = 5, and those given a single dose of pamidronate within 10 days of the burn injury (B+P), *n* = 5. Serum samples were de-identified, meaning that there was no way for the investigators to know or to trace any of the samples to their patient source. Additionally, these samples had been frozen at −80°C since the original study was concluded in 2002.The study was carried out in accordance with the recommendations of approved Protocol 92-304G of the University of Texas Medical Branch Institutional Review Board. All samples were obtained during routine care. Consequently, the Institutional Review Board waived the requirement for written informed consent for the usage of the de-identified samples for research purposes. Written informed consent was obtained from all patients as appropriate and all parents of study participants at the time of enrolling in the approved research protocol. Enrolled subjects were burned ≥40% total body surface area, were predominantly males between the ages of 5 and 18 year, and had normal renal function (11, 13). Serum was obtained from these patients at 4 and 6.5–7 week post-burn. All burned patients had excessive urinary cortisol excretion, up to 8 times the upper limits of pediatric normal values (4, 6) as a consequence of the post-burn stress response.

### Assessment of Myotube Size

Cell layers were fixed in ice-cold acetone-methanol (50:50) and incubated with an anti-Myosin Heavy Chain antibody (MF20, 1:200, Developmental Studies Hybridoma Bank, Iowa City IA) and an AlexaFluor 488-labeled secondary antibody (Invitrogen, Grand Island NY) as described previously (20). Analysis of myotube size was performed by measuring the average diameter of long, multinucleate fibers (*N* = 250–350 per condition) avoiding regions of clustered nuclei on a calibrated tissue image using the Image J 1.43 software (21).

### Western Blotting

Western blots were essentially performed as described previously (20). Total protein extracts were obtained by lysing cell layers in RIPA buffer (150 mMol NaCl, 1.0% NP-40, 0.5% sodium deoxycholate, 0.1% SDS, and 50 mM Tris, pH 8.0) completed with protease (Roche, Indianapolis IN) and phosphatase (Thermo Scientific, Rockford IL) inhibitor cocktails. Cell debris were removed by centrifugation (15 min 14,000 g) and the supernatant collected and stored at −80°C. Protein concentration was determined using the BCA protein assay method (Thermo Scientific, Rockford IL). Protein extracts (30 μg) then underwent electrophoresis in 4–15% gradient SDS Criterion TGX precast gels (Bio Rad, Hercules CA). Proteins were transferred to nitrocellulose membranes (Bio Rad, Hercules CA). Membranes were blocked with SEA BLOCK blocking reagent (Thermo Scientific, Rockford IL) at room temperature for 1 h, followed by an overnight incubation with SEA BLOCK buffer containing 0.2% Tween-20 at 4°C with gentle shaking. After washing with PBS containing 0.2% Tween-20 (PBST), the membrane was incubated at room temperature for 1 h with either Anti-rabbit IgG (H+L) DyLight 800 or Anti-mouse IgG (H+L) DyLight 600 (Cell Signaling Technologies, Danvers MA). Blots were then visualized with Odyssey Infrared Imaging System (LI-COR Biosciences, Lincoln NE). Optical density measurements were taken using the Gel-Pro analyzer software. Antibodies used were pSTAT3-Y705 (#9145), STAT3 (#8768), pAKT-S473 (#4060), AKT (#9272). pERK1/2 (p-p44/42MAPK, T202/Y204, #4370), ERK1/2(p44/42MAPK, #4695), p-mTOR-S2448(#D9C2), mTOR (#7C10), Ubiquitin (#3933), pSmad2-S465/467/Smad3-S423-425 (#27F4), and Smad2/Smad3 (#D7G7) from Cell Signaling Technologies, Danvers MA, LC3B (#L7543) from Sigma-Aldrich, and α-Tubulin (# 12G10) from Developmental Studies Hybridoma Bank (Iowa City IA).

### Statistical Analysis

Results are presented as means ± SD. Significance of the differences was determined by two-way analysis of variance (ANOVA) followed by Tukey's post-test. Differences were considered significant when *p* < 0.05.

## Results

### Effects of Serum From Burned Patients Receiving Either Standard of Care (B) or Standard of Care Plus Pamidronate (B+P) vs. Serum From Normal Unburned Patients (N) on C2C12 Myotubes

**Figure 1:**
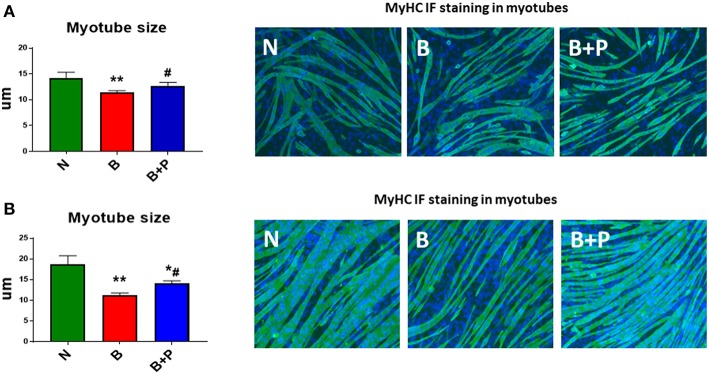
Effects of serum from burned patients receiving standard of care or standard of care and pamidronate on C2C12 myotubes (*N* = 5 per group). Assessment of myofiber size in C2C12 myotube cultures exposed to 1% **(A)** or 5% **(B)** serum for up to 48 h obtained from 3 groups of 5 children: normal unburned (N), burned receiving standard of care after 30 d (B), burned receiving standard of care and pamidronate after 30 d (B+P) (*n* = 250–350 myofibers). Green staining: myosin heavy chain (MyHC). Data (means ± standard deviation) are expressed in micrometers (μm). Significance of the differences: ^*^p < 0.05, ^**^p < 0.01 vs. N, and ^#^*p* < 0.01 vs. B. Significance was determined by two-way analysis of variance (ANOVA) followed by Tukey's post-test.

In order to assess the effects of the serum derived from severely burned children on myotube morphology, fully differentiated C2C12 myotubes were exposed to 1% (Figure 1A) or 5% (Figure 1B) of N, B, or B+P serum. Exposure to B serum caused dose-dependent myotube atrophy compared to N serum (−13%, *p* < 0.01 and −40%, *p* < 0.01, respectively), reproducing the effects of burn injury on muscle atrophy as observed in humans and animals (22, 23). Interestingly, when C2C12 myotubes were exposed to B+P serum, the degree of fiber atrophy was significantly reduced compared to myotubes exposed to B serum (+15%, *p* < 0.05, Figure 1). These experiments were repeated two times.

### Serum Derived From Burned Patients, B and B+P, Induced Pro-atrophic Signaling in the C2C12 Myotubes

**Figure 2:**
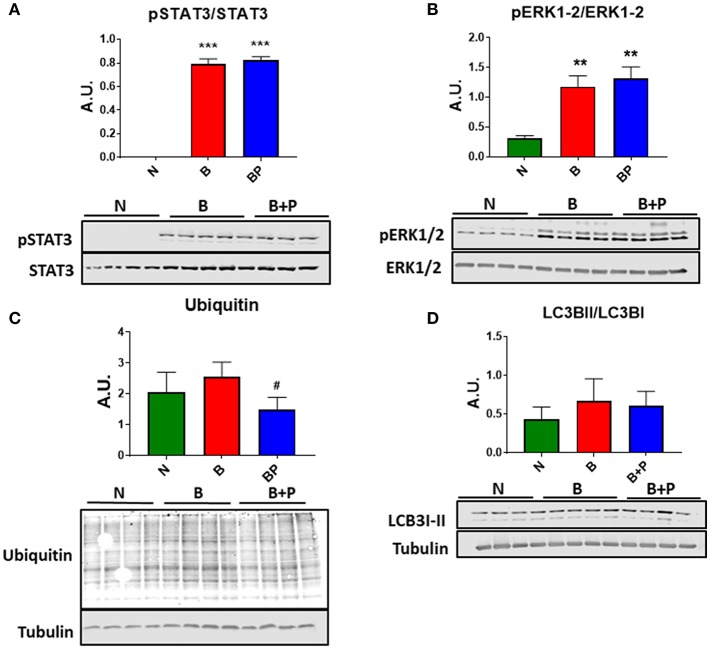
Serum derived from burned patients induced pro-atrophic signaling in the C2C12 myotubes (*N* = 4 per group). Representative Western blotting and quantification of pSTAT3, STAT3 **(A)**, pERK1/2, ERK1/2 **(B)**, Ubiquitin **(C)**, and LC3BII/LC3BI **(D)** in protein extract of C2C12 myotubes exposed for up to 48 h to 5% serum obtained from 3 groups of children: normal unburned (N), burned receiving standard of care after 30 d (B), and burned receiving standard of care and pamidronate after 30 d (B+P). Tubulin was used as a loading control. Data (means ± standard deviation) are expressed as arbitrary units (A.U.). Significance of the differences: ^**^p < 0.01, ^***^p < 0.001 vs. N, and ^#^*p* < 0.05 vs. B. Significance was determined by two-way analysis of variance (ANOVA) followed by Tukey's post-test.

In order to investigate whether the myotube phenotype observed following B or B+P serum exposure was also associated with the modulation of pro-atrophic signaling pathways, the level of proteins associated with protein catabolism was assessed. Interestingly, modulation of several mediators of muscle atrophy were observed using Western blotting analysis performed on whole C2C12 protein extracts. In particular, the activation of the STAT3 signaling pathway (Figure 2A) along with the increase in the pERK1/2/ERK1/2 signaling ratio (+91% B vs. N, +140% B vs. N, *p* < 0.001), both STAT3 and ERK differed from normal by *p* < 0.001 in the myotubes exposed to B and B+P serum (Figure 2B). Interestingly, a non-significant increase of ubiquitin-labeled peptides characteristic of muscle atrophy in the atrophic myotubes exposed to B serum was significantly less in the myotubes treated with B+P serum (−58% vs. B, *p* < 0.05, Figure 2C). This suggests that the partial rescue of myotube size may result from reduced protein catabolism. To investigate the autophagosome-lysosome system, another pathway previously described as being involved in muscle atrophy, the presence of autophagosome accumulation as a marker of autophagy activation was examined. As shown by the ratio of LC3BII/LC3BI protein, no differences in autophagosome accumulation were observed among all three sera (Figure 2D). To assess whether the serum derived from burned patients was also able to induce mitochondrial alterations, we analyzed the expression of proteins related to the control of mitrochondrial homeostasis. The expression of proteins related to mitochondrial biogenesis (PGC 1 α and Cytochrome C) or mitochondrial fusion (OPA1) were unchanged in all experimental conditions (Figure S1). These experiments were performed once.

### Serum From Burned Patients Receiving Pamidronate Normalized Anabolic Signaling Compared to Serum From Burned Patients

**Figure 3:**
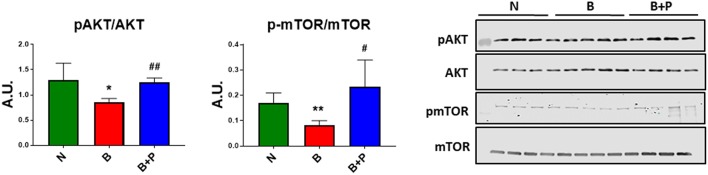
Serum from burn patients receiving pamidronate normalized anabolic signaling compared to serum from burn patients (*N* = 4 per group). Representative Western blotting and quantification of pAKT, AKT, pmTOR, and mTOR in protein extract of murine C2C12 myotubes exposed for up to 48 h to 5% serum obtained from 3 groups of children: normal unburned (N), burned receiving standard of care after 30 d (B), and burned receiving standard of care and pamidronate after 30 d (B+P). Data were normalized to total protein. Data (means ± standard deviation) are expressed as arbitrary units (A.U.). Significance of the differences: ^*^p < 0.05, ^**^p < 0.01 vs. N and ^#^*p* < 0.05, ^##^*p* < 0.01 vs. B. Significance was determined by two-way analysis of variance (ANOVA) followed by Tukey's post-test.

In order to investigate whether B or B+P serum affect protein anabolism in the C2C12 myotubes, the expression of two important markers of anabolism, AKT and mTOR, was quantitated. Myotube atrophy induced by B serum was associated with reduced phosphorylation of AKT (−34% vs. N, *p* < 0.05) and its downstream target mTOR (−51% vs. N, *p* < 0.01), suggesting that muscle anabolism was downregulated through this mechanism (Figure 3). B+P serum was able to restore the phosphorylation of AKT (+46% vs. B, *p* < 0.05) and mTOR (+83% vs. B, *p* < 0.05), thus suggesting that protein anabolism was partially restored in this condition. This experiment was performed once.

### TGFβ Is Responsible for the Effects of Burn Serum on Myotube Size and Pamidronate Rescues Myotube Size Through a Reduction of TGFβ

**Figure 4:**
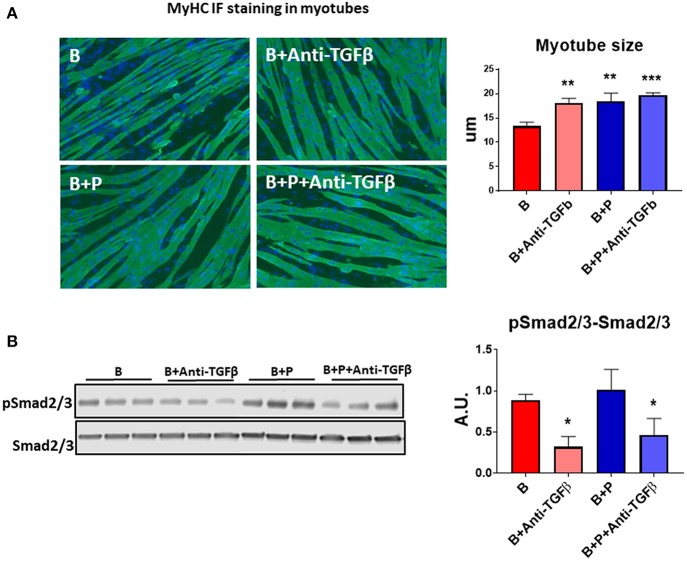
TGFβ is responsible for the effects of burn serum on myotube size (*N* = 3 per group). **(A)** Assessment of myofiber size in C2C12 murine myotube cultures exposed for up to 48 h to 5% serum obtained from 2 groups of children: burned receiving standard of care after 30 d (B), burned receiving standard of care and pamidronate (B+P) after 30 d and co-exposed to a neutralizing antibody, anti-TGFβ-1/2/3. Green staining: myosin heavy chain. Data (means ± standard deviation) are expressed in micrometers (μm). **(B)** Representative Western blotting and quantification of pSMAD 2/3 normalized to total SMAD 2/3 in protein extract of murine C2C12 myotubes exposed for up to 48 h in 5% serum obtained from 2 groups of children: burned receiving standard of care after 30 d (B), and burned receiving standard of care and pamidronate after 30 d (B+P) and co-exposed to a neutralizing antibody anti-TGFβ-1/2/3. Data (means ± standard deviation) are expressed as arbitrary units (A.U.). Significance of the differences: ^*^p < 0.05, ^**^p < 0.01, and ^***^p < 0.001 vs. B. Significance was determined by two-way analysis of variance (ANOVA) followed by Tukey's post-test.

Because TGFβ released from bony metastases due to breast cancer has been implicated in cancer-associated cachexia (19), we chose to evaluate whether TGFβ may be responsible for muscle atrophy following burns. A specific pan-neutralizing antibody, TGFβ-1/2/3 was used. When the myotubes were exposed to B+P serum, their size was significantly larger compared to B serum. Treatment with neutralizing antibody TGFβ-1/2/3 was able to partially protect the myotubes from atrophy when exposed to B serum (+35% vs. B, *p* < 0.001) making the myotube size comparable to those exposed to B+P serum (Figure 4A). To verify the molecular signaling pathway was due to TGFβ, Western blot analysis of the level of Smad phosphorylation was performed. The levels of p-Smad 2/3 were reduced in myotubes treated with the anti-TGFβ-1/2/3 antibody and exposed to either B or B+P serum. No significant difference in Smad phosphorylation was observed in myotubes exposed to B or B+P serum with or without the anti-TGFβ-1/2/3 antibody (Figure 4B). These data suggest that other activators of this pathway may be involved. This experiment was repeated two times.

## Discussion

In this study we used an *in vitro* cell line model to begin to identify directly and indirectly the components of burn serum that are potentially responsible for muscle wasting and to determine the effects of bisphosphonate treatment on their signaling in a model of myotube formation. We found that TGFβ in burn serum is responsible for reduced myotube size and that pamidronate reduces TGFβ activity. Other components of burn serum are inferred via the activation of specific signaling pathways. Highly elevated inflammatory molecules are inferred by the dramatic increase in pSTAT3/STAT3 and pERK1/2/ERK1/2 compared to normal serum. This is also in line with previous experimental evidence reporting that burn injury induces severe muscle wasting and cachexia via a marked increase in pro-inflammatory cytokines (4, 19, 24). Pamidronate treatment appeared to have no effect on factors responsible for these signaling pathways. However, pamidronate did rescue the phosphorylation of AKT and mTOR and reduced protein ubiquitination when compared to burn serum from non-treated patients, suggesting that factors present in burn serum that are responsible for muscle breakdown and catabolism and are prevented from being released by bone due to pamidronate treatment. No effect of pamidronate was observed on the autophagolysosome and mitochondrial gene expression. This *in vitro* study substantiates and extends the initial observations of Borsheim et al. (15) that early use of a bisphosphonate post-burn injury preserves not only bone mass but skeletal muscle mass and strength as well. Moreover, this rescue, initially seen at 4 weeks post-burn, is sustained for at least 6–7 weeks. These data suggest that blocking bone resorption prevents the release of pro-atrophic factors from the bone.

Serum from burn patients receiving pamidronate normalized anabolic signaling and reduced muscle protein catabolism compared to serum from burn patients not receiving pamidronate. The use of bisphosphonates mitigates the reduction of the AKT/mTOR signaling pathway and blunts the activation of the catabolic ubiquitin-associated pathways. The reactivation of muscle protein synthesis and the down-regulation of muscle protein catabolism contribute to restore a condi of positive muscle protein balance. These data suggest that mitigating bone resorption also reduces the release of factors that can induce muscle protein catabolism and decrease muscle protein anabolism (15).

Extensive burn injury is characterized by a release of pro-inflammatory mediators such as cytokines, glucocorticoids, and reactive oxygen species (24, 25). Altogether, these factors are responsible for local and systemic derangements and can drive the bone and muscle alterations that are frequently observed in burn patients. For example, the strong activation of the STAT3 pathway can result from circulating IL-6 in burn serum, in line with previous observations in an experimental model of burn-induced muscle wasting (24) and in human patients (4). This event, in turn, increases overall catabolism and results in muscle wasting as previously described in models of cancer cachexia (20, 26). Interestingly, exposure of C2C12 myotubes to B+P serum did not prevent the increase in STAT3 phosphorylation. These findings suggest that although pamidronate was able to modulate inflammation in a model of multifocal osteomyelitis (27), overall it did not seem to interfere with the systemic inflammation associated with burn injury.

Muscle atrophy after burn injury is also characterized by the downregulation of anabolic signaling (24). In line with these findings, in the present study we reported a reduction of the AKT and mTOR signaling upon exposure of myotubes to serum from burn patients. Consistent with our results, conditions associated with high inflammatory response showed a negative regulation of protein synthesis through the dysregulation of the mTOR/p70S6K axes (28–31). In particular, the cytokines IL-6 and TNFα were previously shown to be directly involved in the inhibition of AKT/mTOR pathways (30, 31). Pamidronate treatment appears to reduce the effect of cytokines involved in AKT and mTOR signaling but not STAT3/ERK. We know that pamidronate can reduce inflammatory cytokines and RANKL (32) in bone and thus may reduce not only TGFβ release from bone, but possibly also other muscle catabolic factors. We know also that other mediators belonging to the IL-6 superfamily, including IL-11 and OSM are present in the serum of animals with burn-induced cachexia and can activate the STAT3 signaling system in muscle (24). However, whether pamidronate can modulate these levels remains unknown.

**Figure 5:**
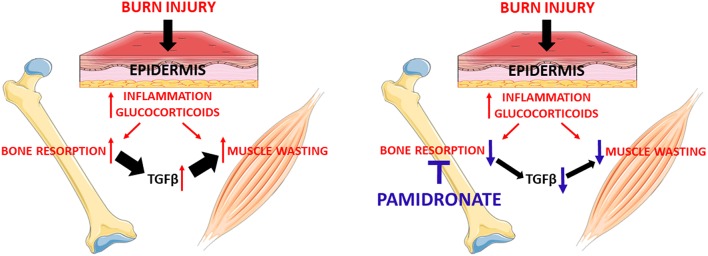
Administration of pamidronate after burn injury through preventing bone resorption hypothetically reduces the release of the TGFβ family members from the bone. The reduction of TGFβ family members seems to be responsible for the partial rescue of myotube atrophy. This signaling appears to be due to the reactivation of the anabolic AKT/mTOR pathway and the reduction of the catabolic ubiquitin-dependent pathway.

TGFβ blockade of burn serum restored C2C12 myotube size to that of pamidronate treated patients. While no effect was observed using the pan-TGFβ antibody on burn serum from pamidronate treated patients on myotube size, pamidronate treatment partially rescued myotube size compared to burn injury with no pamidronate treatment. The magnitude of the myotube size rescue was similar between the effects of the anti-TGFb antibody and the pamidronate treatment. We interpret these data as consistent with pamidronate prevention of release of TGFβ from bone matrix as shown in a hypothetical diagram in Figure 5. Furthermore, we showed that this neutralizing antibody was able to protect the myotubes from undergoing atrophy in the presence of B serum mainly by downregulating the Smad-dependent signaling. However, we could not show a direct connection between the magnitude of Smad signaling and the magnitude of inhibition of myotube formation. Despite the anti-TGFβ-downregulation of Smad phosphorylation, Smad phosphorylation remaining elevated with the pamidronate treated burn serum. A possible explanation for this is that there was still sufficient TGFβ remaining in burned serum to stimulate Smad phosphorylation. Furthermore, we could speculate that other substances present in burn serum, such as myostatin/GDF8 (33) might maintain phosphorylation of Smad. However, we did not measure myostatin in our serum samples. The improvement of myotube size could also be the result of modulation of the AKT/mTOR pathway. Indeed, the myostatin/TGFβ signaling pathway was shown to inhibit the anabolic AKT signaling by means of Smad 2/3 activation, as reviewed by Egerman and Glass (3, 34). Ours is not the first evidence suggesting that blockade of the TGFβ/Smad 2/3 pathways preserves the muscle phenotype in conditions normally associated with muscle wasting. Indeed, administration of ACVR2B/Fc, a synthetic decoy peptide and inhibitor of the signaling downstream of the binding of TGFβ family ligands to the activin receptor type 2B was able to potently preserve muscle mass and prolong survival in a model of cancer cachexia (35). Similarly, we have recently shown that the same inhibitor was able to completely prevent the loss of bone and muscle mass in animals chronically exposed to the chemotherapy regimen Folfiri (36).

Altogether, our results suggest that the anti-resorptive properties of pamidronate prevent the release from the bone of TGFβ-1/2/3 (13) and that the latter may be a catabolic factor that promotes myotube atrophy, thus validating a putative role of TGFβ in promoting muscle atrophy during burn injury. It is unclear whether there are additional muscle catabolic factors within the bone matrix, but the fact that TGFβ is elevated following burns (16–18) and that it has been shown to modulate muscle cachexia in breast cancer patients (19) would suggest that it may at least be one candidate muscle catabolic factor that is liberated from bone following burn injury. The presence of a putative TGFβ mechanism in two such disparate groups of patients as metastatic breast cancer and pediatric burns raises the possibility that this mechanism could be active in a wider range of resorptive bone diseases or may contribute to a cycle of muscle wasting and resorptive bone loss in neuromuscular diseases. The potential role of bisphosphonates or other anti-resorptives should be studied in these situations.

### Shortcomings of the Study

Our interpretation of the data has to be tempered by the variable sample size. Studies were conducted depending on the amount of sample required for each experiment and the remaining sample volume available. However, randomization of the original study should have controlled for potential imbalances between the burn groups. Also, both burn groups B and B+P experienced increased endogenous glucocorticoid production as do all severely burned patients (4, 6) which may have contributed to the reduced myotube size as glucocorticoids impact both muscle wasting and bone turnover. However, the significant differences between the two burn groups would indicate that there is still a partial rescue of myotube size with pamidronate treatment. In addition, while we have previously provided evidence that serum concentration of TGFβ increases in burned patients and animals compared to normal unburned controls, we did not measure the concentration of TGFβ in the samples tested in this study.

## Data Availability

All datasets generated for this study are included in the manuscript and/or the Supplementary Files.

## Ethics Statement

Institutional Review Board of the University of Texas Medical Branch protocol #92-304G. This is the approval of the original randomized controlled double blind prospective study from which the de-identified specimens were obtained for the currently submitted *in vitro* study.

## Author Contributions

LB conceived and designed the experiments. AB and FP designed the experiments. FP performed the experiments. GK conceived the overall idea for the study. FP, AB, LB, and GK wrote and edited the paper. All authors contributed to the writing and editing of the manuscript.

### Conflict of Interest Statement

The authors declare that the research was conducted in the absence of any commercial or financial relationships that could be construed as a potential conflict of interest.

## Supplementary Material

Figure S1 Mitochondrial environment is not affected by serum derived from burned patients (*N* = 4 per group). Representative Western blotting and quantification of PGC1α, OPA-1, and Cytochrome C in a protein extract of murine C2C12 myotubes exposed for up to 48 h to 5% serum obtained from 3 groups of children: normal unburned (N), burn receiving standard of care after 30 d (B), and burn receiving standard of care and pamidronate after 30 d (B+P). Tubulin was used as the loading control. Data (means ± standard deviation) are expressed as arbitrary units (A.U.). Significance was determined by two-way analysis of variance (ANOVA) followed by Tukey's post-test. Differences were considered significant when *p* < 0.05.
