# Supplementary figures and images for: Molecular Mechanisms Responsible for the Rescue Effects of Pamidronate on Muscle Atrophy in Pediatric Burn Patients
Source: Front Endocrinol (Lausanne). 2019 Aug 7;10:543. doi: 10.3389/fendo.2019.00543 (PMC6692456; doi:10.3389/fendo.2019.00543)

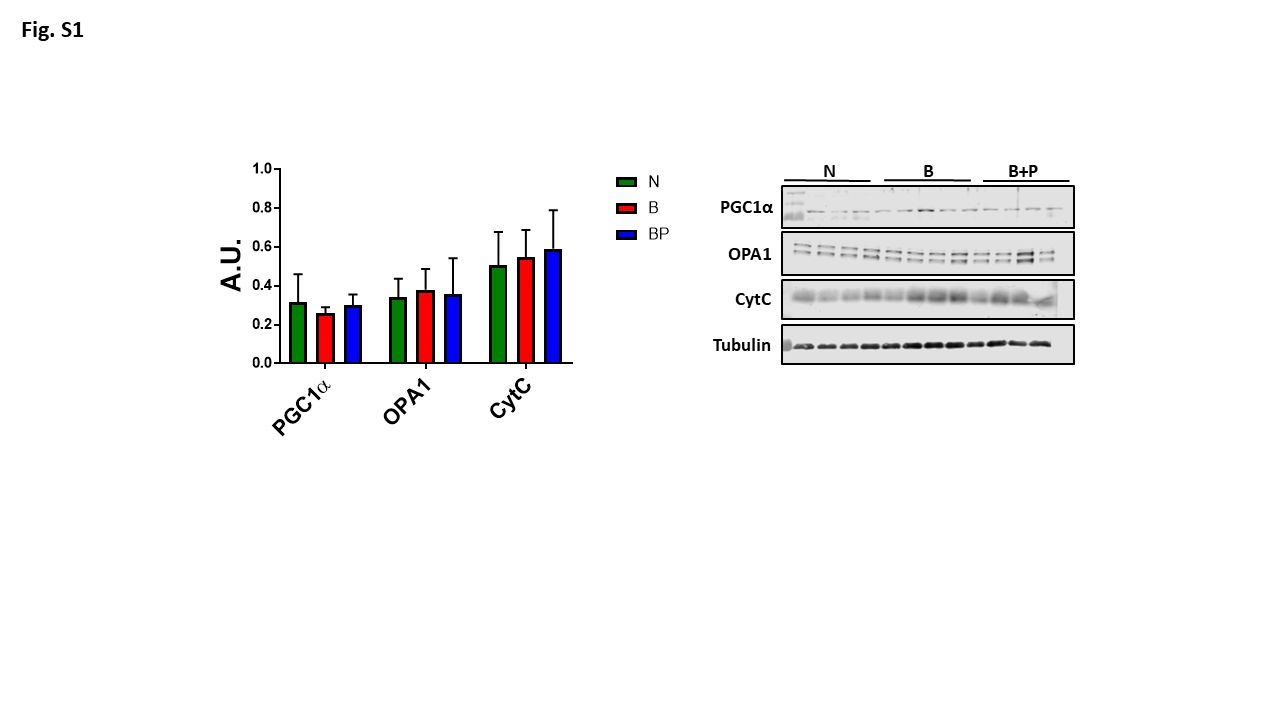

Supplement: Figure S1 — Mitochondrial environment is not affected by serum derived from burned patients (N = 4 per group). Representative Western blotting and quantification of PGC1α, OPA-1, and Cytochrome C in a protein extract of murine C2C12 myotubes exposed for up to 48 h to 5% serum obtained from 3 groups of children: normal unburned (N), burn receiving standard of care after 30 d (B), and burn receiving standard of care and pamidronate after 30 d (B+P). Tubulin was used as the loading control. Data (means ± standard deviation) are expressed as arbitrary units (A.U.). Significance was determined by two-way analysis of variance (ANOVA) followed by Tukey's post-test. Differences were considered significant when p < 0.05. [file Image_1.TIF]
